# Primary gamma-herpesviral infection in Zambian children

**DOI:** 10.1186/1471-2334-10-115

**Published:** 2010-05-12

**Authors:** Veenu Minhas, Brad P Brayfield, Kay L Crabtree, Chipepo Kankasa, Charles D Mitchell, Charles Wood

**Affiliations:** 1 Nebraska Center for Virology and School of Biological Sciences, University of Nebraska-Lincoln, Lincoln, NE 68583 USA; 2 Department of Paediatrics and Child Health, University Teaching Hospital, Lusaka, Zambia; 3 Department of Pediatrics, University of Miami School of Medicine, Miami, FL, 33133 USA

## Abstract

**Background:**

HHV-8 is closely related to Epstein-Barr virus (EBV), but the clinical presentations of these two infections in early childhood are not well understood. Also, it is not known whether infection by one virus correlates with another. Here, we compare the natural history of infection by these two viruses along with the clinical manifestations and risk factors that are associated with early childhood infection in Zambia, which is an endemic area for HHV-8.

**Methods:**

This study was conducted in a cohort of 12 month old Zambian children (N = 677). Data on socio-economic status and a wide range of clinical manifestations were collected. Logistic regression was used to test for significant associations between the collected variables and HHV-8 or EBV serostatus at 12 months of age.

**Results:**

We observed a significantly higher seroprevalence for EBV (58.9%) as compared to HHV-8 (13.4%). HIV-1 infected children had at a significantly higher risk of being infected with HHV-8 (Odds ratio [OR] 3.69, 95% confidence interval [CI] 1.64 - 8.32). HIV-1 infection of the mothers was a significant risk factor for increased acquisition of EBV but not HHV-8 by children (OR 1.86, 05% CI 1.20 - 2.87). Self reported rash was marginally associated with primary infection for HHV-8 and EBV.

**Conclusions:**

These results suggest that there is no correlation between EBV and HHV-8 infections. Infection by one does not increase the susceptibility for the second virus. Primary HHV-8 and EBV infection in early childhood may clinically present as rash but remains largely asymptomatic and may remain undetected in this population. HIV infection in the mother or child are important risk factors that contribute to EBV or HHV-8 infection.

## Background

Human herpesvirus-8 (HHV-8), also known as Kaposi's sarcoma-associated herpesvirus (KSHV) is a gamma-herpesvirus reported to be associated with all forms of Kaposi's sarcoma (KS) [1,2]. HHV-8 is closely related to Epstein-Barr virus (EBV), the only other known human gamma-herpesvirus with both having a latent and lytic phase of replication. EBV is the most commonly identified virus associated with AIDS-related non-Hodgkin lymphomas, CNS lymphomas, African Burkitt's Lymphoma, and Nasopharyngeal Carcinoma [3,4]. HIV-associated immunosuppression has led to an increase in the incidence of childhood Kaposi's sarcoma and non-Hodgkin lymphomas including African Burkitt's lymphoma in sub-Saharan Africa [5].

Little is known about the acquisition of HHV-8 and EBV in early childhood in endemic African countries like Zambia especially after the onset of the HIV-1 epidemic in this region. Whether infection by one virus enhances the susceptibility to infection by the other in the context of HIV-1 infection and immune-suppression is not known. Most studies that documented the prevalence of HHV-8 have sampled a cross-section of the population. These studies have reported various factors that are associated with increased risk for HHV-8 prevalence but not on primary HHV-8 infection and the associated clinical symptoms. In addition, it is still not clear whether primary infection with HHV-8 produces an acute clinical syndrome and what factors increase the risk of acquisition of HHV-8 and EBV. Only one study conducted on immunocompetent children in Egypt has reported that primary HHV-8 infection may present itself as a febrile illness and the infected children developed a maculopapular rash [6]. Whether similar clinical symptoms are observed in endemic areas such as Zambia and whether symptoms are similar for primary HHV-8 and EBV infections is still not known. As a part of our previous cohort studies in Zambia, we recruited newborn infants and followed them for up to 48 months of age. We have found that approximately 40% of children became infected with HHV-8 by 48 months of age [7]. Our cohort provided us an opportunity to further investigate early childhood infection in children less than 12 months of age. We anticipate that children of this age group will most likely undergo primary infection rather than reactivation of latent infection. Due to the prospective design and data collection during follow-up, our study provides a unique opportunity to examine potential risks with these two herpesviruses in the same population.

The objective of this study was to determine HHV-8 and EBV seroprevalence in the children, the effect of HIV-1 infection, the specific clinical manifestations and the risk factors involved. Here, we report the prevalence of HHV-8 and/or EBV serostatus in 12 month old Zambian children where primary infection event had occurred. We also report the clinical symptoms during the primary infection, the association of primary infection with the HIV-1 status of the child and analyze the association of a positive serostatus at 12 months of age with markers of social and economic status.

## Methods

### Study participants

**Figure 1:**
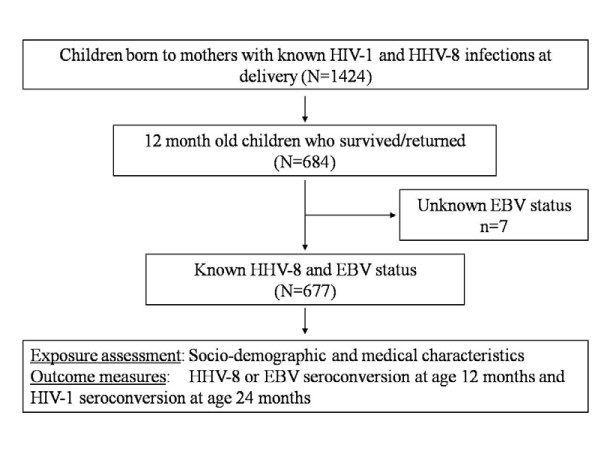
**Flowchart of study cohort**. Flowchart summarizing the longitudinal cohort followed to study herpesviral infection in children in Lusaka, Zambia, 1998-2004. The present analysis includes 677 children who survived/returned at 12 months of age in order to establish reliable HHV-8, EBV and HIV-1 diagnosis.

Enrollment and follow-up- As described previously [7], study participants were recruited as part of a prospective cohort of mother/child pairs at the University Teaching Hospital (UTH) in Lusaka, Zambia between October 1998 and April 2004. Women in the early stages of labor and their newborn children were enrolled in a prospective cohort study after the mothers were counseled, educated about the study, and had given written informed consent. The study was approved by the Institutional Review Boards of the University of Zambia, University of Nebraska, and University of Miami. A total of 1,424 mother-child pairs who returned for at least one post-partum visit constituted our longitudinal cohort (Figure 1).

#### Inclusion and exclusion criteria

Among the cohort of 684 children of 12 months of age, adequate amount of plasma for reliable testing for HHV-8 and EBV was not available for 7 children. Therefore, the present analysis includes 677 children who survived and were followed up till at least 24 months of age in order to establish reliable HIV-1 status. Children who did not return for a follow-up visit at age 24 months (n = 740) were excluded from this analysis. Other reasons for exclusion included early mortality, early withdrawal, and loss to follow-up before HIV-1 serostatus could be reliably established. Diagnostic PCR testing for HIV-1 in children below 18 months of age was not available in Zambia at the time of this study. Therefore, HIV-1 serology was performed between 18 - 24 months of age. By 24 months of age, 6 percent (41/677) of the children tested positive for HIV-1.

### Serological testing

Blood specimens collected from children were coded by a unique identification number and analyzed without knowledge of the personal identity of the study participants. To rule out detection of transplacental maternal HHV-8 antibodies, child plasma was not screened for HHV-8 infection until the age of 12 months and for HIV-1 infection until the age of 18 months.

#### HHV-8 serology

All plasma samples were tested by monoclonal-enhanced immunofluorescence assay (mIFAs) as described previously [8]. Briefly, BC3 mIFAs were conducted by stimulating BC-3 cells with tetradecanoyl phorbol acetate (TPA), which were then fixed with 4% paraformaldehyde and permeabilized after 72 hours. To reduce subjectivity in observing specific fluorescence, slides were read independently by two laboratory workers. All plasma determined to be positive by BC-3 mIFA was further confirmed using Sf9 mIFA. Recombinant baculoviruses expressing glutathione S-transferase tagged lytic proteins, ORF65 and K8.1A, and latent protein, ORF73 were used to develop Sf9 mIFAs. Baculovirus-infected Sf9 cells expressing GST alone were used as a negative control to detect background and nonspecific fluorescence. All infections were initiated separately, to maximize the level of proteins produced, harvested at 72 hpi and fixed using the BC3 cell method. The procedure of Sf9 mIFA was the same as that for BC3 mIFA. A sample was considered seropositive for HHV-8 only if it was positive at a standard serum dilution of 1:40 for both BC3 and Sf9 mIFA (with at least one antigen). To rule out the detection of residual maternal antibodies, plasma of all HHV-8-seropositive children at 12 months who were born to HHV-8-seropositive mothers was titered at birth, at 6 months, and at 12 months. Primary infection event occurred when previously seronegative children became seropositive at 12 months of age.

#### EBV serology

IgG antibodies against EBV viral capsid antigen (VCA) were detected using a standard Enzyme Linked Immunosorbent Assay kit. (ELISA) (Diagnostic Automation, California). This commercially available kit is based on recombinant purified protein of the VCA complex and has a specificity and sensitivity of 100% as reported by the manufacturer. Plasma was diluted 1:21 as per the manufacturer's instructions, and three calibrators and control sera were tested in each test run. The optical density was read on a microplate reader (Biotek instruments) at 450 nm. Results were accepted when all the quality control criteria were met as per the manufacturer's recommendation.

#### HIV-1 Test

All plasma was screened by a standard HIV-1 test kit (Capillus HIV-1/2 Agglutination test kit, Trinity Biotech.) and confirmed for HIV-1 antibodies by another standard kit (Abbott Determine HIV-1/2 EIA, Abbott Laboratories).

### Statistical Analysis

Data was analyzed using the statistical software package, SPSS version 17 (SPSS, Chicago, IL). Logistic regression was used to test for significant associations between the collected variables and serostatus at 12 months of age. Univariate test of association was performed, and odds ratio and 95% confidence intervals (CI) were calculated. We also tested for interactions and multicollinearity by checking the correlation between various variables. Multivariate analysis included all variables that had a p-value ≤ 0.05 to control for possible confounders and identify independent associations.

## Results

Study participants and prevalence of infection: For this report, evidence of seroconversion for HHV-8 and EBV was determined for 677 children when they reached 12 months of age. However, of these 677 children, 655 children were also followed up at least once or more at 2, 4 or 6 months of age also. The remaining 22 children were followed up directly at 12 months of age after enrollment at birth. While analyzing factors associated with seroconversion; 'ever' denotes that the child experienced that condition at least once or more during the 12 month follow-up period and 'never' denotes that the child was never reported to experience that condition during any follow-up visit. Follow-up information was collected when the patient visited the clinic or by active follow-up at the place of residence. At each follow-up visit, trained nurses administered a structured questionnaire in order to obtain information about the socio-economic status, demographic data and medical history of the recruited patients. The questionnaire data collected and analyzed for this report is based on all prominent published studies on HHV-8 prevalence and incidence. Data on socio-demographic factors, ailments, illnesses or symptoms experienced, and information on any medications or treatments received since the last visit were recorded during these visits.

**Figure 2:**
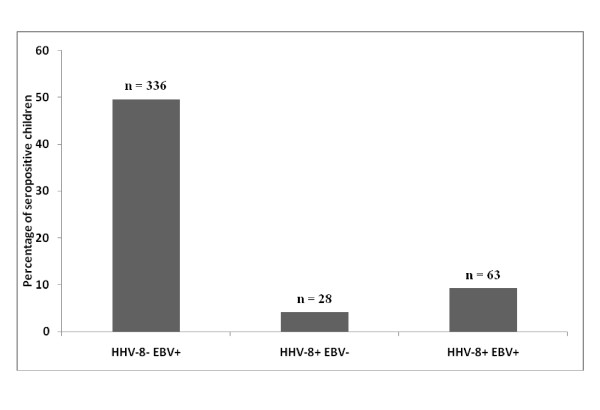
**Prevalence of HHV-8 and EBV infection**. Bar graph depicting the prevalence of HHV-8 and EBV positive children in 12 month old children (N = 677) in Lusaka, Zambia, 1998-2004.

In the cohort of 12 month old Zambian children (N = 677), the prevalence of HHV-8+ EBV- children was 4.14%. We also observed that in this cohort the seroprevalence of EBV to be significantly higher than HHV-8. The prevalence of HHV-8- EBV+ children was 49.63% demonstrating that in this population a significantly larger number of children were infected with EBV alone as compared to HHV-8 alone (Figure 2). Prevalence of dually infected (HHV-8+ EBV+) children was 9.30%.

Primary HHV-8 infection and associated clinical manifestations: Associations between primary HHV-8 infection and a range of socio-demographic characteristics and medical characteristics indicative of the general health of the child were evaluated (Table 1). In a univariate analysis, a self-reported history of rashes was significantly associated with primary HHV-8 infection (OR 1.84, 95% CI - 1.13-3.01). Also, children who were HIV-1 seropositive at 12 months of age (OR 4.79, 95% CI - 2.44-9.38), were infected with EBV or were born to HIV-1 infected mothers had increased odds of primary HHV-8 infection as compared to those who were not. Multivariate analysis indicated that HIV-1 seropositive status (at 18 months of age) was independently associated with primary HHV-8 infection (OR 3.69, 95% CI - 1.64-8.32). After adjusting for confounders, we also observed a weak association between history of rashes in children with primary HHV-8 infection (OR 1.54, 95% CI- 0.92-2.56) but no association with EBV infection. No other self-reported clinical symptoms or socio-demographic characteristics were significantly associated with primary HHV-8 infection. These included self reported history of all common medical conditions (fever, diarrhea, cough, malaria, conjunctivitis, oral thrush) and indirect measures of the health of the child (use of antibiotics, hematinics, multivitamins and administration of traditional therapy).

**Table 1:** Risk factors associated with primary HHV-8 infection.

		**Unadjusted Analysis**		**Adjusted Analysis^b^**	
	**Positive/Total No.^a^**	**p-value**	**OR (95% CI)**	**p-value**	**OR (95% CI)**
			
					
***Socio-Demographic Characteristics***					
					
**Gender**					
- Male	41/347	0.20	0.75 (0.48 - 1.17)		
- Female	50/330		Reference		
**Ethnic Group**					
- Nyanja	45/308	0.17	1.47 (0.85 - 2.56)		
- All others	25/167	0.19	1.52 (0.82 - 2.82)		
- Bemba	21/202		Reference		
**Mothers Education**					
- 0-7 years	45/348	0.69	0.91 (0.59 - 1.42)		
- 8 or more years	46/329		Reference		
**Mothers Employment**					
- Unemployed	78/566	0.60	0.85 (0.45 - 1.59)		
- Employed	13/109		Reference		
**Monthly Income**					
- less than 100,000 ZMK	42/309	0.92	0.66 - 1.59)		
- more than 100,000 ZMK	49/368		Reference		
**Household members**					
- 6 or more	32/247	0.80	0.94 (0.59 - 1.50)		
- 2 to 5	57/418		Reference		
**HIV infection (mother)**					
- Positive	30/149	0.01	1.93 (1.19 - 3.12)	0.66	1.14 (0.63 - 2.08)
- Negative	61/528		Reference		Reference
					
***Child Characteristics^c^***					
					
**Birth weight of child**					
- less than 3.2 Kg	53/430	0.24	0.76 (0.49 - 1.20)		
- more than 3.2 Kg	38/244		Reference		
**Diarrhea**					
- Ever	36/238	0.32	1.26 (0.80 - 1.99)		
- Never	54/436		Reference		
**Fever**					
- Ever	38/270	0.65	1.11 (0.71 - 1.74)		
- Never	52/404		Reference		
**Cough**					
- Ever	52/371	0.58	1.14 (0.73 - 1.78)		
- Never	38/303		Reference		
**Rashes**					
- Ever	28/143	0.01	1.84 (1.13 - 3.01)	0.10	1.54 (0.92 - 2.56)
- Never	62/531		Reference		Reference
**Malaria**					
- Ever	11/78	0.84	1.07 (0.54 - 2.12)		
- Never	79/596		Reference		
**HIV infection (child)**					
- Positive	16/41	<0.001	4.79 (2.44 - 9.38)	0.002	3.69 (1.64 - 8.32)
- Negative	75/636		Reference		Reference
**EBV infection**					
- Positive	63/399	0.03	1.67 (1.04 - 2.69)	0.14	1.45 (0.89 - 2.36)
- Negative	28/278		Reference		Reference
**Antibiotics**					
- Ever	68/475	0.26	1.34 (0.81 - 2.24)		
- Never	22/199		Reference		
**Traditional therapy**					
- Yes	12/61	0.13	1.68 (0.86 - 3.30)		
- No	78/613		Reference		
**Multivitamin use**					
- Ever	7/32	0.15	1.89 (0.79 - 4.50)		
- Never	83/642		Reference		

OR- Odds Ratio, CI- Confidence Interval, ZMK- Zambian Kwacha.

^a^Total number may not add up to 677 in all categories due to missing values.

^b^Adjusted analysis includes all variables with p-value ≤ 0.05 in the unadjusted analysis.

^**c**^Data on lymphadenopathy, pneumonia, conjunctivitis, oral thrush and hematinics use in the child were also analyzed but not shown in this table.

Unadjusted and adjusted odds ratios to predict association between primary HHV-8 infection in a cohort of 677 children and socio-demographic characteristics and medical conditions in Lusaka, Zambia, 1998-2004.

Primary EBV infection and associated clinical manifestations: We also compared the prevalence of primary EBV infection and associated clinical characteristics in the same cohort of 12 month old Zambian children (Table 2). We observed that in a univariate analysis, self reported rash was also associated with EBV infection in children at 12 months (OR 1.56, 95% CI 1.06-2.3). Also, other factors such as fever in the child, low education of mother and HIV infection in the child or mother were individually associated with EBV infection at 12 months. Upon multivariate analysis, we observed that low education of the mother and HIV infection in mother were individually associated with EBV infection (OR 1.51, 95% CI 1.11-2.06 and OR 1.86, 95% CI 1.20-2.87, respectively). As observed with HHV-8, no other self-reported clinical symptoms (rashes, fever, diarrhea, cough, malaria, conjunctivitis, and oral thrush), indirect measures of the health of the child (use of antibiotics, hematinics, multivitamins and administration of traditional therapy) and socio-demographic characteristics were significantly associated with primary HHV-8 infection.

**Table 2:** Risk factors associated with primary EBV infection.

		**Unadjusted Analysis**		**Adjusted Analysis^b^**	
			
	**Positive/Total No.^a^**	**p-value**	**OR (95% CI)**	**p-value**	**OR (95% CI)**
					
***Socio-Demographic Characteristics***					
					
**Gender**					
- Male	199/347	0.39	0.87 (0.64 - 1.19)		
- Female	200/330		Reference		
**Ethnic Group**					
- Nyanja	182/308	0.88	1.03 (0.72 - 1.47)		
- All others	99/167	0.87	1.04 (0.68 - 1.57)		
- Bemba	118/202		Reference		
**Mothers Education**					
- 0-7 years	222/348	0.01	1.51 (1.11 - 2.06)	0.006	1.56 (1.14 - 2.13)
- 8 or more years	177/329		Reference		Reference
**Mothers Employment**					
- Unemployed	331/566	0.56	1.13 (0.74 - 1.72)		
- Employed	61/109		Reference		
**Monthly Income**					
- less than 100,000 ZMK	189/309	0.28	1.18 (0.87 - 1.61)		
- more than 100,000 ZMK	210/368		Reference		
**Household members**					
- 6 or more	148/247	0.74	1.06 (0.77 - 1.45)		
- 2 to 5	245/418		Reference		
**HIV infection (mother)**					
- Positive	108/149	<0.001	2.15 (1.44 - 3.2)	0.005	1.86 (1.20 - 2.87)
- Negative	291/528		Reference		Reference
					
***Child Characteristics^c^***					
					
**Birth weight of child**					
- less than 3.2 Kg	251/430	0.72	0.95 (0.69 - 1.29)		
- more than 3.2 Kg	147/244		Reference		
**Diarrhea**					
- Ever	140/238	0.98	0.99 (0.72 - 1.37)		
- Never	257/436		Reference		
**Fever**					
- Ever	168/270	0.15	1.26 (0.92 - 1.72)		
- Never	229/404		Reference		
**Cough**					
- Ever	214/371	0.48	0.89 (0.66 - 1.22)		
- Never	183/303		Reference		
**Rashes**					
- Ever	96/143	0.02	1.56 (1.06 - 2.3)	0.08	1.43 (0.96 - 2.13)
- Never	301/531		Reference		Reference
**Malaria**					
- Ever	48/78	0.61	1.13 (0.7 - 1.84)		
- Never	349/596		Reference		
**HIV infection (child)**					
- Positive	63/91	0.03	1.67 (1.04 - 2.69)	0.14	1.44 (0.88 - 2.36)
- Negative	336/586		Reference		Reference
**EBV infection**					
- Positive	33/41	0.01	3.04 (1.38 - 6.69)	0.27	1.64 (0.67 - 3.90)
- Negative	41/636		Reference		
**Antibiotics**					
- Ever	273/475	0.24	0.82 (0.58 - 1.15)		
- Never	124/199	Reference			
**Traditional therapy**					
- Yes	40/61	0.27	1.37 (0.79 - 2.37)		
- No	357/613		Reference		
**Multivitamin use**					
- Ever	20/32	0.67	1.17 (0.56 - 2.44)		
- Never	377/642		Reference		

OR- Odds Ratio, CI- Confidence Interval, ZMK- Zambian Kwacha.

^a^Total number may not add up to 677 in all categories due to missing values.

^b^Adjusted analysis includes all variables with p-value ≤ 0.05 in the unadjusted analysis.

^c^Data on lymphadenopathy, pneumonia, conjunctivitis, oral thrush and hematinics use in the child were also analyzed but not shown in this table.

Unadjusted and adjusted odds ratios to predict association between primary EBV infection in a cohort of 677 children and socio-demographic characteristics and medical conditions in Lusaka, Zambia, 1998-2004.

## Discussion

Very little is known about the clinical manifestations associated with primary infection with HHV-8 in early childhood and factors that may be associated with primary infection or an increase in risk of infection, especially in HIV-1 infected children, in an endemic region such as Zambia. The majority of the seroprevalence studies are cross-sectional in design, making it difficult to detect primary infection. Both HHV-8 and EBV are oncogenic gamma-herpesviruses that have a similar mode of transmission, most likely through saliva [9,10]. Therefore, the major aim of this study was to investigate primary HHV-8 and EBV infection, compare the clinical manifestations associated with primary infection and whether they have a similar mode of transmission and whether there is an association between HHV-8 and EBV infection. Detailed analysis of these could provide important information regarding the patterns of transmission, natural history of infection and the susceptible population at higher risk for infection.

Our study has several strengths. We were able to analyze a well characterized cohort of children and their caregivers. We also collected a wide range of data in the form of questionnaires that were administered at each follow-up visit. These questionnaires collected information on most common symptoms and illnesses and any information that may indirectly depict a poor health status of the child. This study was conducted on children from an established cohort of mother-child pairs recruited at the University Teaching Hospital, the affiliated teaching hospital for the University of Zambia School of Medicine in Lusaka, Zambia to study the epidemiology and transmission of HHV-8 in a population that is an endemic area for HHV-8 infection. Our results clearly show that children are infected by both HHV-8 and EBV very early in life. HHV-8 and EBV have been reported to have similar modes of transmission, via saliva. Interestingly, our results show that the incidence of EBV early in childhood is significantly higher as compared to HHV-8. A very similar seroprevalence rate has been reported for EBV in some Asian countries such as India, where Venkitaraman et al have reported that IgG antibodies to VCA rose rapidly to 90% by 5 years of age [11]. The prevalence of VCA-specific IgM and the geometric mean titer of VCA-specific IgG antibodies were highest between the ages of 6 months and 2 years, the median age of primary infection being 1.4 years. Primary infection in Malaysian children reportedly occurs at 4-6 months. Thus the transmission for EBV is either more effective or occurring at a much higher rate as compared to HHV-8, possibly as a result of more frequent exposure. Indeed, a recent study from Uganda has reported that the detection of EBV DNA in saliva was more frequent than HHV-8 DNA [12].

Both EBV and HHV-8 have been reported to have similar modes of transmission via salivary contact, low socioeconomic status and overcrowded living conditions have been considered as a risk factor for acquiring both HHV-8 and EBV. A study conducted on Ugandan children found a direct association of HHV-8 infection with low socio-economic status and the use of surface water [13]. In our study socio-demographic information was collected at birth and data on recent medical history provided by the caregiver, which may directly or indirectly help in assessing the health of the child, were collected at each follow-up visits. We did not observe any association of primary infection with HHV-8 with the reported socio-economic characteristics, including monthly income, mother's educational level, household size or ethnicity. However, direct comparison of different HHV-8 serological studies can be difficult sometimes because of different serological tests that are used for diagnosis of HHV-8 infection, the difference in the age of the children and study cohort. As expected, we did observe that primary infection with EBV was significantly increased in children whose mothers had 7 years or less of education. However, assessing socio-economic status is complex and our questionnaire was not designed to examine direct markers of socio-economic status, therefore the collected information can only provide indirect information about the socio-economic status of the household.

We also analyzed a range of child characteristics that may be associated with HHV-8 and EBV infection in children, and found that HIV-1 infection was found to be a major risk factor. Our earlier studies have found that HIV-1 infected children were more likely to be infected with HHV-8 [7]. Interestingly, unlike HHV-8 we did not find HIV-1 infection risk to be risk factor for EBV infection and we did not find a correlation between EBV and HHV-8 infection in this group of children. These results also show that in the case of EBV the immune status of the child may not be an important factor and that most children in this cohort, regardless of their HIV-1 status, will get infected early in life. In fact, by 2 years of age, we observed that the seroprevalence for EBV was 92% in the same cohort (data not shown). It is possible that for HHV-8, the immune status of the child plays a more significant role while in case of EBV it is the status of the transmitters that is more important. Multivariate analysis showed that HIV-1 infection in mothers was associated with higher risk of EBV infection in children as compared to children born to HIV-1 uninfected mothers. This is most likely because the HIV-1 positive mothers are more likely to have higher EBV viral loads and thus shed more virus in their saliva as compared to HIV-1 negative mothers [14]. Another study, conducted in Canada, has reported similar results where children born to HIV-1 infected mothers seroconverted earlier for EBV, than children born to HIV-1 uninfected mothers [15]. Lack of availability of HAART at the time of this study may have resulted in a high level of transmission of HIV-1 to children but its impact on HHV-8 and EBV transmission is hard to estimate. It is clear that these viruses interact with HIV that predisposes HIV-infected individuals to malignancies. In general, while the risk of developing non-Hodgkins lymphomas in AIDS patients in Africa is lower as compared to United States, it is thought to be associated with severe immunosuppression, poor prognosis and shorter mean survival of the African patients [16-18].

Our data suggests that children who were reported to have had rashes were more likely to seroconvert for HHV-8. Presence of rashes was marginally associated with infections with both HHV-8 and EBV. In fact, primary HHV-8 infection has been reported to be associated with the presence of maculopapular rash [6]. In our study the association was not as strong but it could have been due to difference in population sampling. In the study reported by Andreoni et al children admitted to the emergency ward were sampled which could have biased the study towards observing more severe cases [6]. In our study the follow-up questionnaire did not distinguish between various kinds of rashes; therefore, it is difficult for us to determine an association with a specific type of rash. Overall, appearance of rashes may be indicative of primary infection with either one or both of these infections and a larger sample size would be necessary to examine this association further. Malaria is also endemic in Zambia and we did not find any association between seroconversion for either EBV or HHV-8 with a recent history of malaria. While malaria may not be a risk factor for seroconversion to either virus, a recent study has suggested that EBV and malaria may act synergistically in the pathogenesis of childhood Burkitt lymphoma [19], most likely through the direct reactivation of the virus [20,21].

Our study has several limitations. Firstly, reporting or recall bias leading to exposure misclassification could not be entirely ruled out. In a majority of the cases, the children came back with the caregivers multiple times during the 12 month period. This allowed us to document clinical symptoms at regular intervals. But 22 children came directly at 12 months after their enrollment at birth. This could have led to an underreporting of clinical symptoms from which a child may have suffered. Secondly, it has been reported by Mbulaiteye et al that households with limited access to water may increase the risk for HHV-8 infection [13]. Whether it increases the risk for HHV-8 or EBV primary infection in early childhood could not be analyzed in the present study because this data was not collected. Third, we cannot exactly pinpoint as to when the primary infection event for HHV-8 or EBV took place during the 12 month period. Therefore, it is difficult to argue that associated symptoms could be attributed entirely to either EBV or HHV-8 seroconversion. The number of children who were seropositive for HHV-8 or EBV alone was small to draw decisive conclusions. Lastly, we cannot rule out that the associated symptoms were entirely due to primary seroconversion and not due to reactivation of HHV-8. However, based on our previous study it is likely that children under 12 months of age are usually undergoing primary HHV-8 infection.

While a large percentage of children will eventually become infected by EBV and as we have reported earlier that there is a steady increase in HHV-8 infection in early childhood with age in Zambia, the impact of these infections on the population in the context of HIV-1 infection is not clear. It is possible that the infection by one or both of the viruses may increase the susceptibility to HIV-1 infection or vice versa, as we have observed a significant co-relation between HIV-1 and the two viruses. It is possible that dual infection may affect the disease progression because it has been reported that HHV-8 and EBV may interact at the molecular level and may promote the establishment of latency [22]. Therefore, a better understanding of the interaction between these viruses may lead to the development of better strategies to manage these infections and also in prevention of infection in early childhood.

## Conclusions

Taken together these results suggest that there is no correlation between primary HHV-8 and EBV infection. Primary infection with HHV-8 may clinically present as rash but remains largely asymptomatic in early childhood in Zambia. Because of mild and non-descript symptoms associated with primary infection of HHV-8, it may remain undetected or untreated in this population. Our results also suggest that HIV-1 infection of the mother may be associated with increased risk of EBV infection for the child. HIV-1 infected children are significantly more likely to acquire HHV-8 infection as compared to HIV-1 uninfected children.

## Competing interests

The authors declare that they have no competing interests.

## Authors' contributions

VM contributed to conceptual design, data cleaning, interpretation, and preparation of the final manuscript. BPB contributed to conceptual design, and preparation of the manuscript. KLC participated in the implementation of the study. CK participated in study design and data collection. CDM contributed to study design and drafting of the manuscript. CW contributed to overall scientific management, interpretation of data and writing of manuscript. All authors read and approved the final manuscript.

## Pre-publication history

The pre-publication history for this paper can be accessed here:

http://www.biomedcentral.com/1471-2334/10/115/prepub
